# Establishing a Foundation for the In Vivo Visualization of Intravascular Blood with Photon-Counting Technology in Spectral Imaging in Cranial CT

**DOI:** 10.3390/diagnostics14141561

**Published:** 2024-07-19

**Authors:** Anna Klempka, Philipp Neumayer, Alexander Schröder, Eduardo Ackermann, Svetlana Hetjens, Sven Clausen, Christoph Groden

**Affiliations:** 1 Department of Neuroradiology, University Medical Centre Mannheim, Medical Faculty Mannheim, University of Heidelberg, 68167 Mannheim, Germany; 2 Department of Medical Statistics and Biomathematics, Medical Faculty Mannheim, University of Heidelberg, 68167 Mannheim, Germany; 3 Department of Radiation Oncology, University Medical Centre Mannheim, Medical Faculty Mannheim, University of Heidelberg, 68167 Mannheim, Germany

**Keywords:** photon-counting CT, intravascular imaging, intracranial blood, spectral imaging, non-enhanced imaging

## Abstract

Background: Advances in computed tomography (CT) technology, particularly photon-counting CT (PCCT), are reshaping the possibilities for medical imaging. PCCT in spectral imaging enables the high-resolution visualization of tissues with material-specific accuracy. This study aims to establish a foundational approach for the in vivo visualization of intracranial blood using PCCT, focusing on non-enhanced imaging techniques and spectral imaging capabilities. Methods: We employed photon-counting detector within a spectral CT framework to differentiate between venous and arterial intracranial blood. Our analysis included not only monoenergetic +67 keV reconstructions, but also images from virtual non-contrast and iodine phases, enabling detailed assessments of blood’s characteristics without the use of contrast agents. Results: Our findings demonstrate the ability of PCCT to provide clear and distinct visualizations of intracranial vascular structures. We quantified the signal-to-noise ratio across different imaging phases and found consistent enhancements in image clarity, particularly in the detection and differentiation of arterial and venous blood. Conclusion: PCCT offers a robust platform for the non-invasive and detailed visualization of intravascular intracranial blood. With its superior resolution and specific imaging capabilities, PCCT lays the groundwork for advancing clinical applications and research, notably in the diagnosis and management of intracranial disorders. This technology promises to improve diagnostic accuracy by enabling more precise imaging assessments.

## 1. Introduction

In recent years, advancements in computed tomography (CT) imaging technology have once again revolutionized diagnostic capabilities within the field of medical imaging [1]. Among these innovations, photon-counting technology, equipped with novel detectors, stands out as a significant development for enhancing visualization [2]. Better understanding of the promising material decomposition capabilities of CT is needed in order to find purposeful clinical application. We are dealing with an increased amount of data, of which analysis can contribute to a better utilization of physiology and pathology with PCCT [3,4]. Research into the spectral properties and tissue decomposition capabilities of photon-counting CT has already begun, although primarily focusing on in vitro studies. These studies highlight sensible directions for development and advanced possibilities for differentiating substances [5].

This study aims to establish a foundation for the in vivo visualization of intracranial blood using photon-counting technology, which offers material discrimination capabilities not available with the energy-integrated detectors of traditional CT. Our research material, blood, plays a pivotal role in many pathological processes, and due to the heterogeneity of blood forms, it is an intriguing material to characterize in imaging [6]. The intravascular blood characterization, differentiating between arterial oxygenated and venous non-oxygenated blood, could support information about hemorrhages or extravasation and their sources. Identification of the source of blood, whether arterial or venous, could lead to a differentiation of intracranial bleeding types such as perimesencephalic bleeding, which is estimated to be venous in comparison to subarachnoid hemorrhage. Further use could aid in assessing the age of the bleeding with a simple and fast PCCT scan. In this context, we see the potential of our work, as well as the practical application and clinical integration of photon-counting CT for the spectral imaging of intravascular blood in vivo. The problem of material decomposition can be analyzed thanks to significant advancements in CT imaging technologies. The evolution of CT includes the introduction of spiral CT, followed by multi-slice CT, wide-cone CT, dual-source CT, dual-energy CT, and, most recently, photon-counting CT (PC CT) [1]. Commonly used energy-integrating detectors (EIDs) rely on a detector bank converting X-ray radiation into visible light. Following this conversion, the photons of visible light are absorbed by a photodiode composed of semiconductor material. Then, the emitted energy from this photodiode is proportional to the total energy within a measurement interval, rather than reflecting the individual energy of each X-ray photon [2].

In contrast, PC CT bypasses the conversion into visible light. Initially, a voltage is applied between the cathode atop the semiconductor layer and the anode below. When X-rays strike these layers, they release electron pairs that migrate toward the anode. These pairs induce short current pulses, which are then transformed into voltage pulses through a pulse-shaping circuit. The pulse height is proportional to the total energy of the absorbed X-ray radiation. Once the pulses surpass a certain threshold, they are counted [3]. While electric noise is a common occurrence in EID CTs, as with all other electrical devices, it can be effectively eliminated in PC CTs by setting the reference voltage sufficiently high [4]. An advantage lies in artifact reduction (e.g., in Thorax CT), stemming from the PC CT’s capability to discern individual photons based on their energy levels [7]. With the same radiation exposure, PC CTs produce less image noise, thereby enabling a simultaneous reduction in radiation dosage. Additionally, the resolution of PC CTs is superior, as the pixel electrode size is significantly smaller than that of EID CTs’ detector elements. Additional functionalities include better virtual monoenergetic imaging and enhancements in the virtual non-contrast image.

The innovations now evident in the structure and properties of imaging offer new avenues for what is known as spectral imaging. These opportunities stem from the enhanced technical capabilities introduced by the new photon-counting (PC) detector. Utilizing these advances in clinical medicine requires a precise clinical definition of the properties of the substances under investigation in order to accurately assess their so-called density or gray scale level in Hounsfield units (HUs). Some authors have described the so-called decomposition of certain substances, for example, Risch F. et al. [8]. Computer tomographic spectral information can also be collected with dual-energy CT. Dual-energy CT (DECT) has already enabled material differentiation using two different X-ray energy levels, which facilitates differentiation based on atomic number and specific material characteristics. Current technologies such as MRI as well as sonographic imaging distinguish blood vessels based on flow.

What needs to be addressed is the fact that some substances, such as blood in the human body, which are critically important exhibit different properties depending on whether they are examined ex vivo or in vivo. The appearance varies depending on its location within tissues or outside of them in the form of hematomas, active bleeding, or due to the state of oxygenation relative to arterial or venous blood. For blood, DECT can generate virtual non-contrast images and iodine maps, aiding in blood assessment without contrast agent interference, but this technique is associated with a higher radiation dose than in diagnostic cranial CT [9]. Because of this, the use of PC CT would be preferred.

In PC CT, in terms of imaging techniques, virtual non-contrast (VNC) imaging [10], iodine mapping, virtual monoenergetic, and traditional polyenergetic imaging serve unique diagnostic purposes. VNC, which can be derived from dual energy or PCCT data, eliminates the need to expose patients to additional scans without contrast, reducing the radiation dose and enhancing patient safety. Iodine mapping, facilitated by spectral imaging, precisely locates and quantifies iodine contrast within tissues, providing some insight in studies [11]. In imaging modalities like VNC and iodine mapping, it is customary to use HU to quantify the density of tissues and materials. However, it is essential to acknowledge that HU values can vary with the kV settings or reconstruction kernel of the imaging system. This variation requires calibration and the careful interpretation of HU values, especially when comparing data across different imaging settings or modalities.

Therefore, the aim of our study is to determine the level of density in in vivo blood imaging within the human body, focusing on the PCCT of asymptomatic patients. Density measurements, introduced with the advent of the first CT detectors, have proven useful in identifying pathologies and recognizing physiological stages. Blood in vessels typically exhibits varying densities, measured in HU. Normally, the density of blood ranges from approximately 30 to 45 HU when not enhanced by contrast [12]. We focus on non-enhanced imaging to effectively characterize intravascular intracranial blood. The circulation pathway of oxygenated blood is relatively short as it travels from the heart, through the aortic arch, into the cervical and intracranial arterial vessels. Venous blood, returning from the brain and head structures, accumulates in the venous sinuses before returning to the heart through cervical venous vessels [13,14,15]. The detection of intracranial intravascular blood can provide additional information and assist in differentiating spectral decomposed values of pathology, for example, hemorrhages or extravasation.

While there are studies that explore these capabilities with PC CT, our research takes a different approach by examining the physiological spectrum of intravascular blood and establishing a foundational understanding of physiological statuses. This foundation will support further studies of pathology, such as hemorrhages, extravasations, or even blood clots [16]. There is already potential in imaging to determine the age of hemorrhages or blood clots by detecting their density. With PC CT, we introduce a novel dimension to image reconstruction by capturing new spectral information. This capability allows for more sophisticated material decomposition, enhancing differentiation between materials at a molecular level and potentially better-identifying lesions.

## 2. Materials and Methods

### 2.1. Patients

The institutional review board granted approval (number 2024-811) for conducting a retrospective analysis. We analyzed non-enhanced cranial CT scans performed on a PC CT between 1 January 2024 and 23 December 2021. The inclusion criteria were the absence of intra-axial pathology, no history of intra-axial surgery, no implanted foreign bodies such as extra ventricular drains, and no history of blood diseases. We specifically excluded any patients with recent histories of bleeding or intracranial surgery to ensure our sample reflected a homogeneous patient population in terms of baseline cranial characteristics. This approach minimized potential variability in readings caused by post-surgical changes or the presence of acute hemorrhagic components. From all patients examined with this PC CT, we identified 90 cranial CT scans from 75 patients with a median age of 76.5 years (SD ± 13.25), including 25 females and 50 males.

### 2.2. Spectral Image Acquisition

All scans were conducted using a cranial CT protocol on the PC CT scanner, Naeotom Alpha by Siemens Healthineers (Forchheim, Germany). Settings included 120 kV, quality reference 72 mAs, ME67, pitch factor 0.35, rotation time 0.5 s, and matrix size 512 × 512, using spiral acquisition. Each patient included in the study underwent a standardized cranial CT protocol that utilized consistent radiation parameters to maintain uniformity in imaging quality and reproducibility, and the spectral imaging data set was always present.

### 2.3. Spectral Evaluation

**Figure 1:**
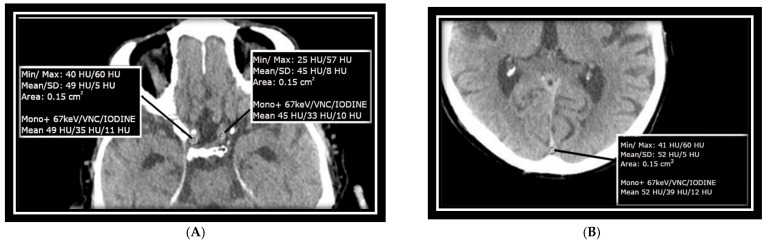
Axial non-enhanced CT scans of the neurocranium, highlighting two key areas: (**A**) bilateral ICA (internal carotid arteries): measurements were taken to capture the dimensions and structural integrity of the internal carotid arteries on both sides. (**B**) Confluence of sinuses: focus was placed on this region to assess the venous confluence at the base of the skull. Each measurement was conducted with meticulous attention to detail, ensuring that the measurements strictly adhered to the anatomical boundaries of the vessels and avoided any measurement errors on the vessel walls.

Measurements were taken at the confluence of sinuses (CS) over an area of approximately 15 mm^2^, as well as at the transitions between the left and right internal carotid arteries (ICAs) at the base of the skull in the intracranial part, each with an ROI of approximately 15 mm^2^. These areas were specifically selected to match the size of the ICA/MCA junction and to avoid calcified plaques that could impair measurement accuracy. The mean value of HU as well as SD measurements as baseline HU values were recorded from monoenergetic reconstructed data at +67 keV for each selected ROI, with a slice thickness of 1 mm and a soft kernel reconstruction. As shown in other studies and used in the clinical setting, this also is an energy with great discrimination between white and gray matter [17]. Virtual non-contrast (VNC) images and iodine images derived from the same slice of the scan were included as well. Image measurements were conducted using Siemens’ syngo.via software version 8.3 and are presented in Figure 1A,B.

### 2.4. Signal-to-Noise Ratio

To quantify the image quality, we used signal-to-noise ratio (SNR), defined as the ratio of the signal representing the measured target to the background noise inherent in the image. To estimate the SNR, mean signal intensity HU were divided with the SD of the measurements in a monoenergetic +67 keV image setting.

### 2.5. Statistical Analysis

All statistical calculations were performed using SAS software release 9.4 (SAS Institute Inc., Cary, NC, USA). Quantitative parameters, approximately normally distributed, are presented by mean values and standard deviations; for skewed data, median, minimum, and maximum values are given.

## 3. Results

### 3.1. Internal Carotid Arteries

Arterial intracranial intravascular blood corresponds to oxygenated blood. The measurements were conducted with caution to avoid assessing atherosclerotic plaques on the vessel walls. Both the right and left internal carotid arteries (ICAs) demonstrated similar Hounsfield units (HUs), with the right ICA exhibiting a slightly higher value at the 5th percentile. This uniformity in measurements was expected due to the same type of oxygenated blood.

**Figure 2:**
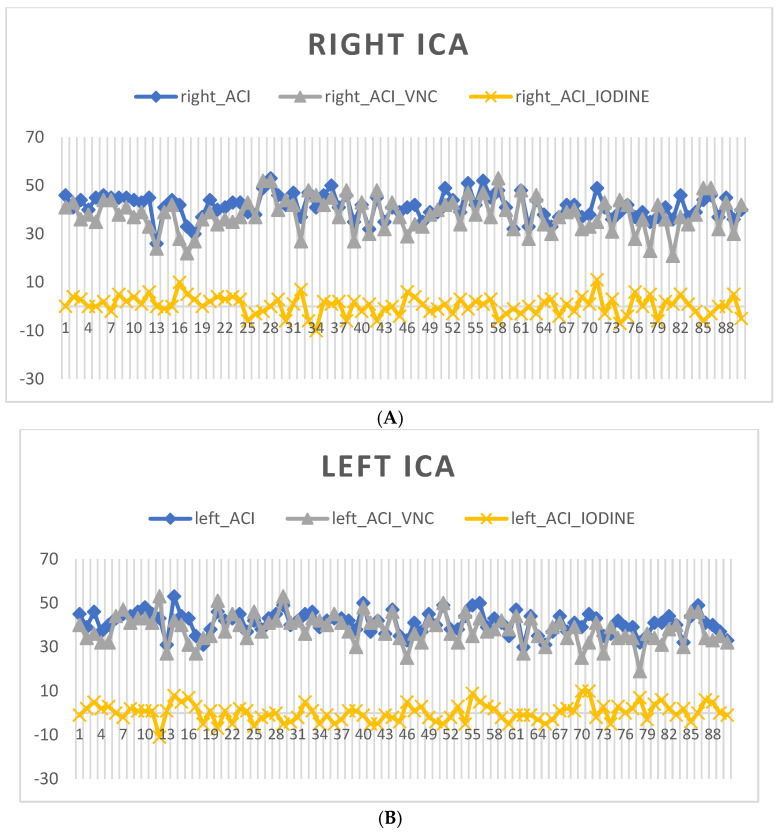
A figure showing arterial blood in blue HU, gray VNC virtual native phase, yellow IODINE phase (**A**) right; (**B**) left ICA (internal carotid artery).

Virtual non-contrast (VNC) measurements for both the right and left ICAs showed consistent values across all percentiles, suggesting uniformity in the non-contrast imaging of these arterial regions. IODINE spectral map measurements for the ICAs displayed negative values at the lower percentiles and single digits at the higher percentiles, as in Figure 2A,B.

### 3.2. Confluence of Sinuses

**Figure 3:**
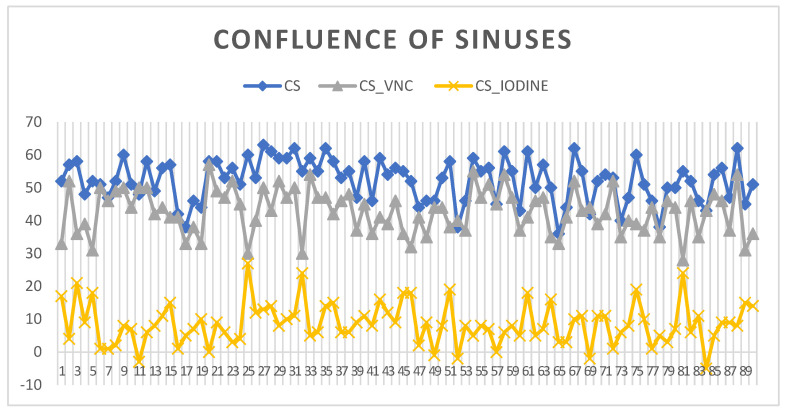
Image showing the measurements of patients’ confluence of sinuses: blue HU at 67 keV, gray VNC virtual native phase, yellow IODINE phase (CS = Confluence of sinuses).

The confluence of sinuses (CS) shows venous intracranial intravascular blood. The HU measurements at this key venous structure within the brain indicated a 5th percentile value of 40 HU, establishing a lower limit of radiodensity for the sample. The 95th percentile reached 62 HU, which remains within the normal range for non-contrast CT measurements of blood. The standard deviation values for the confluence of sinuses suggest a consistent radiodensity with a narrow spread from 3 at the 5th percentile to 7 at the 95th percentile, indicating minimal variation in HU measurements across the sample. The VNC values for the CS show that the lower 5% of measurements are at 31 HU, while the upper 95% reach 54 HU, reflecting the expected range without the influence of contrast material. IODINE spectral imaging values in this study range from slightly negative at the 5th percentile to 19 at the 95th percentile, as seen in Figure 3.

Comparing the measurements in ICAs and CS, the results highlight the distinct characteristics between the CS and ICAs in terms of radiodensity and iodine phase measurements. The CS consistently shows higher HU values across all measurements, indicating a denser structure in the venous area compared to the arterial regions of the ICA. The ICA shows similar distributions between the right and left sides, with slight variations that might be of clinical interest. The distributions (5th, 10th, 90th, and 95th) for various variables across a sample of 90 individuals show as visible in Table 1 and are described in detail.

### 3.3. Monoenergetic +67 keV HU Measurements

Higher HU measurements across all percentiles compared to both the right and left ICAs were seen in CS. This indicates a generally higher radiodensity compared to the arterial regions (ICAs). Both sides of the ICA show similar values at each percentile, with the right ICA consistently slightly higher than the left, which is especially noticeable at the lower percentiles (5th and 10th).

### 3.4. Virtual Native Phase

Again, the CS showed higher HU values compared to both the right and left ICA. This consistency across phases underlines the CS’s higher baseline radiodensity. The right ICA showed marginally higher values than the left at the lower percentiles (5th and 10th), which equalized by the 90th and 95th percentiles. This could suggest minor variations in the composition or physical properties of the arteries on each side.

### 3.5. Iodine Phase

The values for CS started at a negative value at the 5th percentile, gradually increasing to 19 HU at the 95th percentile. This progression indicates a range of detection of iodine, from nearly none to a moderate level, reflecting typical venous characteristics. Both arteries started with negative values, suggesting minimal iodine presence or measurement anomalies at the lower percentiles.

### 3.6. SNR as Image Quality Control

The SNR for the CS images was measured at 11.8 with a standard deviation of ±4.1. This relatively high SNR indicates good image quality, enabling clear visualization of the venous confluence structures, which is essential for accurate diagnosis and assessment. The SNR for the right ICA was 9.4 with a standard deviation of ±3.5, while the left ICA recorded an SNR of 8.8 with a standard deviation of ±3.6. These values, while slightly lower than those of the CS, still provide sufficient image clarity to effectively evaluate the arterial anatomy and identify pathological changes.

## 4. Discussion

Our study enhances the differentiation possibilities by adding two more values to the initial one already present in a previous scanner measured with Hounsfield unit (HU). The integration of standardizations associated with clinical usage into the development of detectors was a crucial component of our study. Although differences in arterial and venous blood densities are already well-documented within EID research [18], the use of PC CT detectors has introduced additional spectral imaging capabilities without increasing radiation doses (as shown in our pervious study [19]).

One limitation was that our study focused on a retrospective elderly cohort. While it is crucial to acknowledge that laboratory blood values generally do not vary significantly with age due to standardized blood tests, we did not verify the blood tests of our patients, assuming the health of intravascular blood. The primary aim was to establish a foundation for imaging oxygenated and deoxygenated blood, not to compete with laboratory blood analysis. We did not investigate the iodine levels in the blood, which could potentially influence the iodine phase; however, the difference in iodine concentration between venous and arterial blood is not considered a significant clinical aspect. Nonetheless, exploring the capabilities of spectral imaging was a key objective. Another important point to show was that in considering human anatomy, we measured one point in the venous system and two in the arterial system, thus doubling the count of arterial blood measurements. This approach allowed us to collect measurements that could be more influenced by localization and artifacts as well as by blood flow.

Another limitation of our study was the absence of a comparative analysis with other scanners concerning image quality. However, these aspects are comprehensively addressed in our concurrent research [19]. It is already established that, depending on the comparison scanner, PC CT scanners often generate lower radiation exposure in cranial computer tomography. This supports the advantage of obtaining additional clinical spectral information compared to other scanners.

An interesting aspect of our findings was the differences in signal-to-noise ratio [20], which might result from either increased noise or other factors related to blood flow due to arterial pressure with higher volume or the localization of the measured vessels (arterial at the skull base, venous dorsally near the calvaria). The slight variability in measurements of the ICAs on both sides indicates a greater variability than that observed at the CS, potentially reflecting a higher variability in blood density at these locations or influenced by the slight decentral positioning of patients in the PC CT scanner [21]. The SD was relatively close, i.e., the mean signal has the greater impact, which is a positive result meaning that there is not an image quality problem but rather actually different signal strengths.

We showed that spectral photon-counting imaging offers greater differentiation of tissue and can capture all intracranial structures simultaneously, enabling a comprehensive overview in a single scan [17]. This capability not only enhances the efficiency of imaging procedures but also allows for retrospective analysis and recalculations, which can be particularly useful in complex diagnostic cases.

Despite these advantages, there are notable challenges associated with the use of PC CT spectroscopy. The radiation dose remains a concern, as CT scans generally involve higher radiation exposure compared to non-ionizing methods like MRI. Thus, MRI allows some specifications of substances and some aspects such as, for example, spectral imaging is limited and multi-contrast-imaging cannot be performed in MRI.

Our study showed the ability to establish basic parameters for differentiating between intravascular venous and arterial blood based on oxygenation levels—arterial blood being richly oxygenated and venous blood less so. Researching intravascular blood is complex due to its varying constituents as well the continuous blood flow, which can significantly affect imaging results. The differentiation between venous and arterial blood in imaging has been explored to some extent, providing a foundation for further research. Furthermore, our results can be used by estimation of contrast agent accumulation or extravasation.

Looking to the future, this study builds a foundation for the potential to enhance the differentiation capabilities of PC CT spectroscopy, particularly with respect to identifying the age of hemorrhages and differentiating various pathological entities. For example, extravascular blood presents unique challenges, as it undergoes distinct physiological repair processes that can alter its appearance in imaging studies. Future research could focus on measuring blood exsanguination to estimate the arterial or venous filling in arteriovenous malformations using three-dimensional imaging. This would improve the ability to differentiate between hemorrhagic lesions and other fluids, such as ascites, and better identify the extravasation of mixtures between blood types, enhancing diagnostic accuracy and patient outcomes.

Intravascular blood, a primary focus of our research, presents unique challenges due to its varying constituents, which can significantly affect imaging outcomes. Distinguishing between venous and arterial blood types is critical, as they differ primarily in oxygen content. Furthermore, our study contributes to ongoing efforts in distinguishing between hemorrhagic conditions and the presence of a contrast agent, a differentiation crucial for diagnosing conditions like endoleak and extravasation.

## 5. Conclusions

This study underscores PC CT in enhancing the in vivo visualization of intravascular blood within the cranial space. By leveraging advanced photon-counting detectors, we achieved precise differentiation between oxygenated and non-oxygenated blood in spectral imaging, facilitating an improved diagnosis of conditions such as hemorrhages and extravasations without increasing radiation exposure.

The spectral imaging features of PC CT, including virtual non-contrast imaging and iodine mapping, significantly contribute to safer and more detailed assessments, paving the way for their integration into clinical practice. The results confirm that PC CT not only offers superior image quality but also holds the potential to revolutionize diagnostic imaging on the level of discrimination of tissue.

## Figures and Tables

**Table 1:** This table presents a clear differentiation in HU values in the study using percentiles, described more in the text above. Abbreviations: CS: confluence of sinuses, ICA: internal carotid artery. All numbers are in HU (Hounsfield units).

	5th Percentile	10th Percentile	90th Percentile	95th Percentile
Monoenergetic 67keV HU—Measurements				
CS	40	43.5	60	62
ICA right	33	35	47.5	49
ICA left	32	35	47	49
Virtual Native Phase				
CS	31	33	52	54
ICA right	27	28	47.5	49
ICA left	27	30	46	48
Iodine Phase				
CS	−1	1	18	19
ICA right	−6	−6	5	6
ICA left	−5	−5	5	7

## Data Availability

The data presented in this study are available upon reasonable request from the corresponding author.
